# Epidemiologic profile of otorhinolaryngological, head and neck disorders in a tertiary hospital unit in Greece: a challenge for general practitioners?

**DOI:** 10.1186/1472-6815-6-12

**Published:** 2006-06-07

**Authors:** Emmanouil K Symvoulakis, Spyridon Klinis, Athanasios Alegakis, Dionysios E Kyrmizakis, Emmanouil I Drivas, Georgios Rachiotis, Anastas Philalithis, George A Velegrakis

**Affiliations:** 1 Department of Social Medicine, Faculty of Medicine, University of Crete, Greece; 2 Biostatistics Laboratory, Faculty of Medicine, University of Crete, Greece; 3 Department of Otorhinolaryngology, University of Crete, School of Medicine, Greece; 4 Department of Epidemiology and Biostatistics, National School Public Health, Athens, Greece

## Abstract

**Background:**

In Greece, primary care is still developing. The aim of this study was to define the epidemiologic profile of common otorhinolaryngological, head and neck disorders in order to help general practitioners to deal with them in a primary care future.

**Methods:**

A total of 6771 patients attended the Otorhinolaryngology emergency department of the University General Hospital of Heraklion (Crete), between January and December 2004. All cases were included in this retrospective study. The registry of the Otorhinolaryngology emergency department was analysed and age, sex, seasonality and clinical diagnosis were tabulated. All patients were evaluated by Otorhinolaryngologists. The classification of the cases was based on the main symptom or clinical sign that conditioned the reason for seeking care. Diagnoses were also coded according to the International Classification of Diseases (ICD-10).

**Results:**

The male to female ratio was 1:1. The mean age for females was 36.3 years standard deviation (SD): 21.1 and for males was 36.8 years (SD = 22.0). Eight hundred eighty six patients (13.1%) formed the paediatric sub-group. Over 60% of the cases were classified in ten major groups of diagnosis. Acute tonsillitis (12.3%) and acute pharyngitis (9.0%) were the most common causes of all medical visits, followed by otitis media (7.6%) and external ear canal obstruction by ear wax (6.2%). Oedema of the larynx was detected in 0.4%. A negative diagnosis of otorhinolaryngological, head and neck disorder was formulated in 553 patients (8.2%). Hospitalization rate was 5.2%. The highest rate of visits was registered in March.

**Conclusion:**

Most patients used the facility as a primary care service. Real emergencies were a minority. Recovering data about which areas of Otorhinolaryngology deserve more emphasis might help primary health care providers to diagnose and manage the common otorhinolaryngological, head and neck disorders properly.

## Background

Issues of cost have influenced, dramatically, health care delivery in Western countries. General practitioners were encouraged to see and treat a wider spectrum of conditions in order to decrease costs [1].

In Greece, primary care is not fully developed, especially in urban areas. There is a lack of primary care settings and of general practitioners in the cities. People with a variety of disorders attend hospitals or private specialists in order to be reassured, diagnosed and treated [2]. The burden of hospital care is dramatically high and the financial impact is considerable. Additionally, every visit is an isolated episode of health care provision. The lack of care continuity, probably, could trigger further use of care services.

Otorhinolaryngological, head and neck disorders are common causes for seeking care in the emergency department of the University Hospital of Heraklion, Crete. Crete is the biggest island in Greece and the second biggest (after Cyprus) of the East Mediterranean. According to the 2001 census the population of Crete reaches 603.000 inhabitants [3]. The prefecture of Heraklion is the largest of the island, with approximately 295.000 people. There are two public hospital units in the city of Heraklion of which one is the University General Hospital and the other is a secondary level hospital unit. The two hospitals are alternatively on duty every 24 hours, being the duty days for the University hospital approximately 183 per year.

The aim of this study was to define the epidemiologic profile of the common otorhinolaryngological, head and neck conditions in order to help primary care providers to focus on them. In areas where general practice is under development, the medical records from specialised centres could represent the main source of information [4] regarding epidemiologic data. This does not reflect the real community needs but has to be considered when assessing, preliminarily, the health needs of a population.

## Methods

All patients who attended the Otorhinolaryngology emergency department of the University General Hospital of Heraklion, Crete (Greece), between January to December 2004, were included in this retrospective study. From the registry of the Otorhinolaryngology emergency department, data related to the medical history, clinical examination and, whenever necessary, basic laboratory investigations were collected. Otorhinolaryngologists evaluated all patients. Sex, age, seasonality of the visits, most common otorhinolaryngological, head and neck disorders and their frequency were recorded. The clinical diagnosis, used for the classification of the cases, was based on the main symptom or clinical sign that conditioned the reason for seeking care. Diagnoses were also coded according to the International Classification of Diseases (ICD-10) [5]. We did not use the mentioned coding system for groups such as possible precancerous lesions and possible malignant neoplasms.

The total hospitalization rate (%) was extracted. Specific Hospitalization Index was calculated, for the most common otorhinolaryngological, head and neck disorders related to hospitalisation, by dividing the number of the admitted patients in clinic by the number of patients seen at the emergency department, having a specific diagnosis of otorhinolaryngological, head and neck disorder.

Statistical analysis of the data was performed with the package SPSS 13.0. Categorical variables such as sex, seasonality of visits, type of disorder and hospitalisation of the cases were described as counts (n) or proportions (%) while mean, standard deviation (SD) and median values were used for age (continuous variable). Graphical and tabular descriptions were also used to summarise data and present descriptive associations between different variables.

The present study conforms to the principles outlined in the Declaration of Helsinki. In our hospital, no ethical approval was necessary for this kind of retrospective study.

## Results

**Figure 1:**
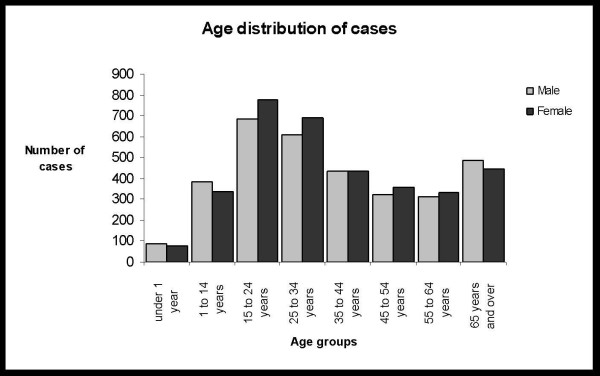
Age distribution of all cases attended the Otorhinolaryngology emergency department.

**Figure 2:**
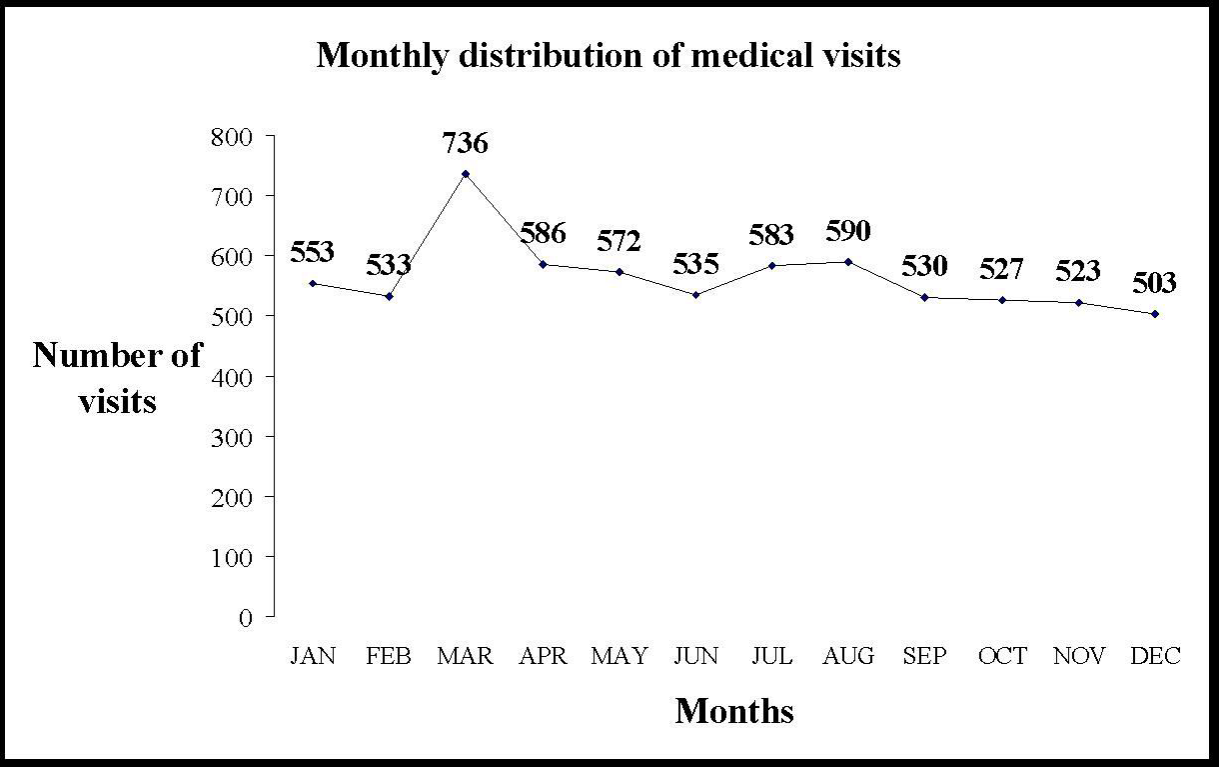
Monthly distribution of all visits in the Otorhinolaryngology emergency department.

A total of six thousand seven hundred seventy one patients (6771) attended the Otorhinolaryngology emergency department. Three thousand four hundred fifty four (51.0%) patients were women and 3317 (49.0%) were men. The mean age for females was 36.3 years (SD: 21.1) and for males was 36.8 years (SD = 22.0). Eight hundred eighty six patients (13.1%) formed the paediatric sub-group. Age distribution of cases is shown in Figure 1. Monthly distribution of cases shows the highest rate of visits (736 or 10.9%) during March, probably due to the increased number of cases with viral infections and allergic symptoms [Figure 2]. The pattern of otorhinolaryngological, head and neck disorders is shown in Table 1. The median age of patients with the ten most common otorhinolaryngological, head and neck disorders is shown in Table 2. From the top ten groups of disorders only dizziness, epistaxis and benign paroxysmal positional vertigo were related to older age groups (patients with median age between 57 and 58 years). The other seven of the ten most common groups were related to younger age groups (patients with median age between 18 and 34 years), [Table 2].

**Table 1:** Distribution of cases by type of disorder.

**Type of otorhinolaryngological head and neck disorder**	**ICD-10**	**Cases (n)**	**Percentage (%)**
Acute tonsillitis	J03	830	12.3
Acute pharyngitis	J02	612	9.0
Acute otitis media	H66.9	516	7.6
External ear canal obstruction by ear wax	H61.2	421	6.2
Diffused or localized external otitis	H60	403	6.0
Acute sinusitis	J01	371	5.5
Dizziness*	R42	236	3.5
Foreign bodies	T17.9, T16	236	3.5
Nasal epistaxis	R04.0	229	3.4
Benign paroxysmal positional vertigo	H81.1	222	3.3
Otomycosis	H62.2	165	2.4
Seasonal allergic rhinitis	J30.1, J30.2	158	2.3
Nose injuries	S00.3, S01.2	156	2.3
Secretory otitis media	H65.9	151	2.2
Various face, and neck injuries**	S00.9	137	2.0
Tinnitus and hearing loss	H93.1, H91.9	124	1.8
Laryngitis	J04.0, J06	120	1.8
Stomatitis	K12	84	1.2
Purulent rhinitis	J31.0	83	1.2
Ear injuries	S00.4, S01.3	72	1.1
Chronic otitis media	H66.3	71	1.0
Facial nerve paralysis	G51	64	0.9
Lymphadenitis	L04.0	63	0.9
Seborrhoic cyst/furuncle	L02	61	0.8
Temporo-mandibular joint syndrome	K07.6	54	0.8
Various acute hypersensitivity reactions***	J39.3	49	0.7
Peritonsillar abscess/phlegmon	J36	46	0.7
Indolent lymph node enlargement	C77.0	39	0.6
Possible precancerous lesions		39	0.6
Inflammation of salivary glands	K11	38	0.6
Infectious Mononucleosis	B27	35	0.5
Possible malignant neoplasms		30	0.4
Oedema of the larynx	J38.4	29	0.4
Sudden hearing loss	H91.2	24	0.4
Others		250	3.7

No clinical findings of otorhinolarynological, head and neck disorders		553	8.2

Total		6771	100

* All subtypes except benign paroxysmal positional vertigo

** Except those of nose and ear

*** Except oedema of the larynx

**Table 2:** The ten most commonotorhinolaryngological, head and neck disorders and median age of the patients

**Top ten groups of otorhinolaryngological, head and neck disorders**	**Median Age (years)**
1. Acute tonsillitis	24.0
2. Acute pharyngitis	31.0
3. Diffused or localised external otitis	34.0
4. Acute otitis media	18.0
5. External ear canal obstruction by ear wax	33.0
6. Acute sinusitis	32.0
7. Dizziness*	57.5
8. Foreign bodies	31.5
9. Nasal epistaxis	58.0
10. Benign paroxysmal positional vertigo	57.0

* except benign paroxysmal positional vertigo

Acute tonsillitis was observed in 12.3% (830 patients), followed by acute pharyngitis (9.0%, 612 patients) and acute otitis media (7.6%, 516 patients). External ear canal obstruction by ear wax accounted for 6.2% (421 patients). Diffused or localized external otitis was observed in 6.0% (403 patients). Acute sinusitis was detected in a percentage of 5.5% (371 patients), followed by dizziness of various aetiology (3.5%, 236 patients) with exclusion of benign paroxysmal positional vertigo, which has been classified as a group apart. Foreign bodies of throat, nose and ear were observed in 3.5% (236 patients) and nasal epistaxis in 3.4% (229 patients). In two hundred twenty two patients (3.3%), Dix-Hallpike manoeuvre was favourable for a diagnosis of benign paroxysmal positional vertigo. The top ten groups of otorhinolaryngological, head and neck disorders accounted for over 60% of all cases while twenty four of low frequency groups of disorders accounted for less of 32% [Table 1]. Of the low frequency groups, possible precancerous lesions were observed in a percentage of 0.6% (39 cases) and possible malignant neoplasms in 0.4% (30 cases).

Oedema of the larynx, per definition a life threatening condition was observed in 29 patients (0.4%). In the group *others *(3.7%) were included all disorder types with a lower frequency than 0.4%, for each subtype (data not shown). No otorhinolaryngological, head and neck disorder was observed in 8.2% (553 patients).

In 10% of the cases, consultations were performed within other specialities in the emergency department. For instance, consultations were performed within internal medicine specialists in 4.02% of the cases, paediatricians in 2.07%, neurologists in 1.28%, chest physicians in 0.68% and surgeons in 0.45%. Consultations, within dentists, accounted for 0.32% of the cases and for the rest of the cases (1.18%) within other specialities.

Hospitalisation rate was accounted for 5.2% (352 patients). The ten most common otorhinolaryngological, head and neck disorders related to hospitalisation with the respective specific hospitalisation index are shown and described in Table 3.

**Table 3:** The ten most commonotorhinolaryngological, head and neck disorders related to hospitalisation and specific hospitalisation index (SHI).

**Top ten otorhinolaryngological, head and neck disorders related to hospitalisation**	**Emergency department visits n**	**Admissions n**	S.H.I** (*range: 0–1*)
Dizziness (all types)*	458	53	0.12
Facial nerve paralysis	64	47	0.73
Peritonsillar abscess/phlegmon	46	44	0.96
Acute tonsillitis	830	31	0.03
Oedema of the larynx	29	29	1.00
Sudden hearing loss	24	23	0.96
Acute sinusitis	371	16	0.04
Nasal epistaxis	229	12	0.05
Various hypersensitivity reactions	49	12	0.24
Indolent lymph node enlargement	39	8	0.21

* including benign paroxysmal positional vertigo

** SHI = Number of admitted patients in clinic/Number of patients seen at emergency department, having a specific diagnosis of otorhinolaryngological, head and neck disorder (in one year).

SHI = 0, none of the patients who attended the emergency department with a specific otorhinolaryngological, head and neck disorder was hospitalised (in one year).

SHI = 1, all the patients who attended the emergency department with a specific otorhinolaryngological, head and neck disorder were hospitalised (in one year).

## Discussion

Otorhinolaryngological, head and neck disorders are among the common reasons of visit in the University General Hospital of Heraklion, the only one tertiary hospital unit in the island of Crete. Adolescents and young adults seem to be the patients who seek care more commonly. Females were slightly more than males. The most common disorder observed was acute tonsillitis. The top ten conditions accounted for over 60% of all cases. Five out of those ten groups are infectious diseases. Perez Obon et al. reported infectious disorders first, among the most frequent conditions, accounting for 41% [6]. Our data suggest that infectious disorders are the leading reason for consultation. One out of two patients used the emergency department service having an infectious disorder.

Dizziness of various aetiology and benign paroxysmal positional vertigo were observed in similar frequencies (3.5% and 3.3%, respectively). It is shown that benign paroxysmal positional vertigo was the cause of seeking care in one out of two patients who presented with a 'dizziness' condition in the emergency department. Nose, ear, and the other injuries accounted for over 5.0%. Various etiologic agents and different types of injuries (skin, soft tissue, vessel or bone lesions) result in different approaches of management. For this reason we reported a breakdown of injury subtypes according to the anatomical position of the noxious impact. Additionally, we cannot estimate, precisely, the total amount of otorhinolaryngological, head and neck injuries; some patients with serious head, chest or abdominal life-threatening traumas and an otorhinolaryngological, head and neck co-injury were evaluated in different speciality settings within the emergency department.

Possible precancerous lesions and possible malignant neoplasms were, initially, diagnosed in a percentage of 0.6% and 0.4% respectively. These observations cannot be confirmed without cytological or histological findings. On the other hand most of the patients with possible precancerous disorders or cancer are seen in the regular Otorhinolaryngology outpatient settings since the onset of their complaints is gradual. A possible overlap with the group of indolent lymph node enlargement represents another point of scepticism.

Oedema of the larynx, per definition, a life threatening condition occurs in 0.4%. Some other conditions as the diagnosis of foreign bodies, nasal epistaxis, injuries, various hypersensitivity reactions or their possible complications, need an urgent or a highly specialized initial medical approach. All these conditions accounted for 13.8%. Timsit et al reported in a similar study that only 10% of the consultations appeared to be real emergencies [7]. It seems that there was a primary care use of the Otorhinolaryngology emergency department facilities, as it has been proposed in other studies as well [8]. Our results suggest that general practitioners could provide a first appropriate contact care for patients with common otorhinolaryngological, head and neck disorders.

Otorhinolaryngological, head and neck diagnosis was negative in a percentage of 8.2%. This fact, perhaps, could be explained by a variety of reasons. Transient and atypical minimal symptoms or somatoform stress reactions could increase consultation rates. Schmidt et al found that the incidence of hypochondriasis was relatively high (13%) among the new patients who attended the Otorhinolaryngology outpatient settings, with frequent overuse of care services and high consumption of drugs [9]. Furthermore the use of a specialized care service per definition cannot provide holistic approaches. Self-referred patients could misuse care services by attending different specialists in order to be sufficiently reassured. This fact maybe explains, partly, the paradox of the patients-non 'otorhinolaryngological, head and neck' patients who attended the Otorhinolaryngology emergency department. Research needs to be done in order to define why more than five hundred fifty persons, in one-year period, asked for care without findings of otorhinolaryngological, head and neck disorders.

The hospitalisation rate was 5.2%. The specific hospitalisation index, as shown in the Table 3, is a pure value and provides information regarding the inpatient or outpatient management of a certain otorhinolaryngological, head and neck disorder. Among the ten most common otorhinolaryngological, head and neck disorders related to hospitalisation, accounting for 78% of the 352 cases, oedema of the larynx, sudden hearing loss, peritonsillar abscess/phlegmon and facial nerve paralysis show the highest specific hospitalisation index. This type of information could be used, as a tool in order to discriminate which conditions should be managed at a secondary care environment.

The limitation of our study consists in the fact that our data are drawn from only one hospital institution. It is difficult to assess the real needs of the community if there is a lack of evidence from primary care settings or without large community-based surveys [10,11]. On the other hand these data largely support the hypothesis that there is use of the hospital services as a primary care facility [12]. By using this information as baseline we could have a preliminary approach regarding the health needs of this population.

## Conclusion

General practitioners should have the skills to deal with the most common disorders. Many of the otorhinolaryngological, head and neck conditions could be managed at the level of primary health care [13]. Retrieving data and incorporating them into the educational and training program of general practitioners might help to achieve successful management of the frequent otorhinolaryngological, head and neck problems in a primary care future. This may decrease the burden of hospital care and improve the quality of care provision in terms of proper referrals, holistic view of patient's condition and continuity of care.

## Competing interests

The author(s) declare that they have no competing interests.

## Authors' contributions

EKS, SK, AP, GAV were involved with the study conception. EKS, SK, DK prepared the manuscript. EKS, SK, AA, GR and EID performed the data acquisition and interpretation. DK, AP and GAV were involved in revising the article for important intellectual content and technical details. All authors read and approved the final manuscript.

## Pre-publication history

The pre-publication history for this paper can be accessed here:


